# Expression and Function of Methylthioadenosine Phosphorylase in Chronic Liver Disease

**DOI:** 10.1371/journal.pone.0080703

**Published:** 2013-12-06

**Authors:** Barbara Czech, Katja Dettmer, Daniela Valletta, Michael Saugspier, Andreas Koch, Axel P. Stevens, Wolfgang E. Thasler, Martina Müller, Peter J. Oefner, Anja-Katrin Bosserhoff, Claus Hellerbrand

**Affiliations:** 1 Department of Internal Medicine I, University Hospital Regensburg, Regensburg, Germany; 2 Institute of Functional Genomics, University of Regensburg, Regensburg, Germany; 3 Institute of Pathology, University of Regensburg, Regensburg, Germany; 4 Grosshadern Tissue Bank and Center for Liver Cell Research, Department of Surgery, Ludwig-Maximilians-University Munich, Munich, Germany; University of Navarra School of Medicine and Center for Applied Medical Research (CIMA), Spain

## Abstract

To study expression and function of methylthioadenosine phosphorylase (MTAP), the rate-limiting enzyme in the methionine and adenine salvage pathway, in chronic liver disease.

**Design:**

MTAP expression was analyzed by qRT-PCR, Western blot and immunohistochemical analysis. Levels of MTA were determined by liquid chromatography-tandem mass spectrometry.

**Results:**

MTAP was downregulated in hepatocytes in murine fibrosis models and in patients with chronic liver disease, leading to a concomitant increase in MTA levels. In contrast, activated hepatic stellate cells (HSCs) showed strong MTAP expression in cirrhotic livers. However, also MTA levels in activated HSCs were significantly higher than in hepatocytes, and there was a significant correlation between MTA levels and collagen expression in diseased human liver tissue indicating that activated HSCs significantly contribute to elevated MTA in diseased livers. MTAP suppression by siRNA resulted in increased MTA levels, NFκB activation and apoptosis resistance, while overexpression of MTAP caused the opposite effects in HSCs. The anti-apoptotic effect of low MTAP expression and high MTA levels, respectively, was mediated by induced expression of survivin, while inhibition of survivin abolished the anti-apoptotic effect of MTA on HSCs. Treatment with a DNA demethylating agent induced MTAP and reduced survivin expression, while oxidative stress reduced MTAP levels but enhanced survivin expression in HSCs.

**Conclusion:**

MTAP mediated regulation of MTA links polyamine metabolism with NFκB activation and apoptosis in HSCs. MTAP and MTAP modulating mechanisms appear as promising prognostic markers and therapeutic targets for hepatic fibrosis.

## Introduction

Impaired expression and function of genes regulating polyamine metabolic pathways have been described in chronic liver disease and these alterations may contribute to the progression of hepatic fibrosis and malignant transformation [1–3]. Methylthioadenosine phosphorylase (MTAP) is the rate-limiting enzyme in methionine and adenine salvage pathways. MTAP catalyzes the phosphorylation of 5'-deoxy-5'-methylthioadenosine (MTA), which is a by-product of polyamine synthesis, to yield adenine and methylthioribose-1-phosphate (MTR-1P). MTR-1P is then converted in a series of enzymatic reactions to regenerate methionine [4]. Furthermore, MTAP regulates polyamine synthesis, as MTA acts an inhibitor of several key enzymes in this pathway [5]. Consistent with its central metabolic role, MTAP expression is high in normal liver tissue [6]. In HCC, on the other hand, loss or downregulation of MTAP leads to accumulation of MTA, which promotes tumorigenicity [7,8].

Chronic liver injury, inflammation, fibrosis and, finally, liver cirrhosis often precede the development of hepatocellular carcinoma (HCC). The activation of hepatic stellate cells (HSCs) is a central event in the development of hepatic fibrosis and, ultimately, cirrhosis. Upon hepatic injury, HSCs transform to an active, highly proliferative myofibroblast-like phenotype that is responsible for excessive matrix deposition in chronically damaged livers [9,10]. The activity of the transcription factor NFκB is increased during HSC activation and induces the resistance of HSCs against apoptosis, which is critical for the development and progression of hepatic fibrosis [11,12]. HSCs also form the HCC stroma and promote tumorigenicity of HCC cells [13–16]. Of note, we have shown that down-regulation of MTAP and consequent elevation of intracellular and extracellular MTA levels further induce the expression of procancerous genes in HSCs [7].

In this study, we assessed the expression and function of MTAP in chronic liver disease. Aside from a downregulation of MTAP expression in hepatocytes of diseased livers, we revealed strong MTAP expression in activated HSCs. Moreover, we observed high MTA levels in activated HSCs compared to hepatocytes indicating that in addition to the lower MTAP expression and concomitant higher MTA levels in hepatocytes also activated HSCs contribute to MTA levels in diseased livers. Loss and gain of function studies confirmed MTAP as critical regulator of MTA levels in activated HSCs. Moreover, we identified MTAP regulated MTA levels as modulator of NFκB activation and apoptosis resistance in activated HSCs. Both promoter methylation and oxidative stress were identified as critical regulators of MTAP expression in activated HSCs. These findings may open new avenues to the prognosis and treatment of hepatic fibrosis and cancer.

## Materials and Methods

### Chemicals

5′-Deoxy-5′-(methylthio)adenosine (MTA), arsenic trioxide (As_2_O_3_), adenosine, periodate oxidized (AdOx), 5-azacytidine (Aza), and N-acetyl-L-cysteine (NAC) were purchased from Sigma-Aldrich Chemie GmbH (Deisenhofen, Germany). Inhibitor of survivin YM155 was purchased from Selleckchem (Munich, Germany).

### Cells and cell culture

Primary human hepatocytes (PHH) and hepatic stellate cells (HSCs) were isolated and cultured as described [17,18]. *In vitro* activation of HSCs was achieved by cell culture on uncoated tissue culture dishes as described [19].

### Experimental murine models of hepatic injury

Bile duct ligation (BDL) or sham operations were performed as described [19]. Mice were fed for 30 weeks either a control or a NASH inducing diet containing 30% lard, 1.25% cholesterol and 0.5% sodium cholate [20,21]. All procedures followed the University of Regensburg guidelines for the care and use of laboratory animals and the Institutional Animal Care and Use Committee approved the study. Liver tissue specimens were either fixed in 10% formalin or snap frozen in liquid nitrogen and stored at -80°C for subsequent analysis.

### Human tissue specimens

Formalin-fixed, paraffin-embedded human liver tissues for immunohistochemical analysis were retrieved from the surgical pathology files of the Institute of Pathology of the University of Regensburg. Human liver tissue for cell isolation and expression analyses was obtained according to the guidelines of the charitable state controlled foundation HTCR (Human Tissue and Cell Research), with the informed patients' written consent, and the study was approved by the local ethics committee of the University Regensburg. Experiments involving human tissues and cells have been carried out in accordance with The Code of Ethics of the World Medical Association (Declaration of Helsinki).

### Quantification of MTA and S-adenosyl-L-methionine (SAM) by LC-ESI-MS/MS

MTA and S-adenosyl-L-methionine levels in cells, tissues and in the supernatant of cultured cells were analyzed by liquid chromatography-electrospray ionization-tandem mass spectrometry (LC-ESI-MS/MS) [7].

### Analysis of mRNA expression

Isolation of total cellular RNA and reverse transcription were performed as described [22]. Quantitative real-time PCR was performed with specific sets of primers applying LightCycler technology (Roche, Mannheim, Germany) as described [22].

### Protein analysis

Protein extraction and Western blotting were performed as described [17,22] using monoclonal anti-MTAP and polyclonal anti-survivin antibodies (both from Abcam, Cambridge, UK; 1:1.000); monoclonal phospho-IκB and phospho-p65 antibodies (Cell signaling Technology, Danvers, USA) were used at a dilution of 1:1.000.

Immunohistochemical analysis of MTAP was performed as described [7]. For immunohistochemical analysis cells were fixed with 4% paraformaldehyde and permeabilized with 0.1% Triton X 100. After blocking (5% BSA), cells were incubated with anti-MTAP antibody (Abcam; 1:200), washed and then incubated with secondary antibody (Cy3 conjugated goat anti mouse IgG (H+L); 1:100; Invitrogen, Darmstadt, Germany). For immunofluorescent costaining deparaffinized sections were blocked with 1%BSA/PBS and incubated with anti MTAP (Abcam 1:200) and anti α-sma (Abcam 1:400) antibody in 10% FCS and 1%BSA/PBS. After washing sections were incubated with Cy2- and TRITC conjugated secondary antibodies (1:200 Dianova, Hamburg, Germany) and a DAPI staining was performed to identify cellular nuclei. Images were collected by fluorescence microscopy using a Zeiss Axioskop2 mot plus microscope (Zeiss, Göttingen, Germany).

### Transfection experiments and luciferase reporter gene assay

Cells were transfected with a MTAP expression plasmid or empty control vector (pcDNA3) using the lipofectamine plus method (Invitrogen) as described [23]. Applying the HiPerFect method (Qiagen, Hilden, Germany), small interfering RNA (siRNA; Hs_MTAP_1,2 HP; all from Qiagen) was transiently transfected into HSCs to deplete MTAP expression as described [7]. The NFκB-luc plasmid (Promega) was used for reporter gene assays. Transfection efficiency was normalized to renilla luciferase activity by cotransfecting 0.1 µg of the plasmid pRL-TK (Promega). All transfection experiments were repeated at least three times.

### Detection of cellular reactive oxygen species (ROS) formation

ROS formation was analyzed with an assay applying cell-permeant 2',7'-dichlorodihydrofluorescein diacetate (H2DCFDA) according to the manufacturer's instructions (Invitrogen). Briefly, cells were incubated with H2DCFDA at a concentration of 100 μM for 30 min at 37°C, and after washing with PBS, ROS formation was detected using a multi-well fluorescence plate reader (Spectra Fluor Plus, Tecan, Männedorf, Switzerland) with excitation and emission filters of 485 and 535 nm, respectively.

### Analysis of apoptosis

Cells were stained simultaneously with FITC-conjugated Annexin V and propidium iodide (both Promokine, Heidelberg, Germany) and analyzed by ﬂow cytometry as described [18]. Further, the Apo-One Homogeneous Caspase-3/7 Assay (Promega) was used to analyze caspase-3/7 activity according to the manufacturer's instructions.

### Statistical analysis

Results are expressed as mean ± standard error (range) or percent. Comparison between groups was made using the Student's unpaired t-test. A p value <0.05 was considered statistically significant. All calculations were performed by using the GraphPad Prism Software (GraphPad Software, Inc., San Diego, USA).

## Results

### MTAP expression in chronic liver disease

**Figure 1:**
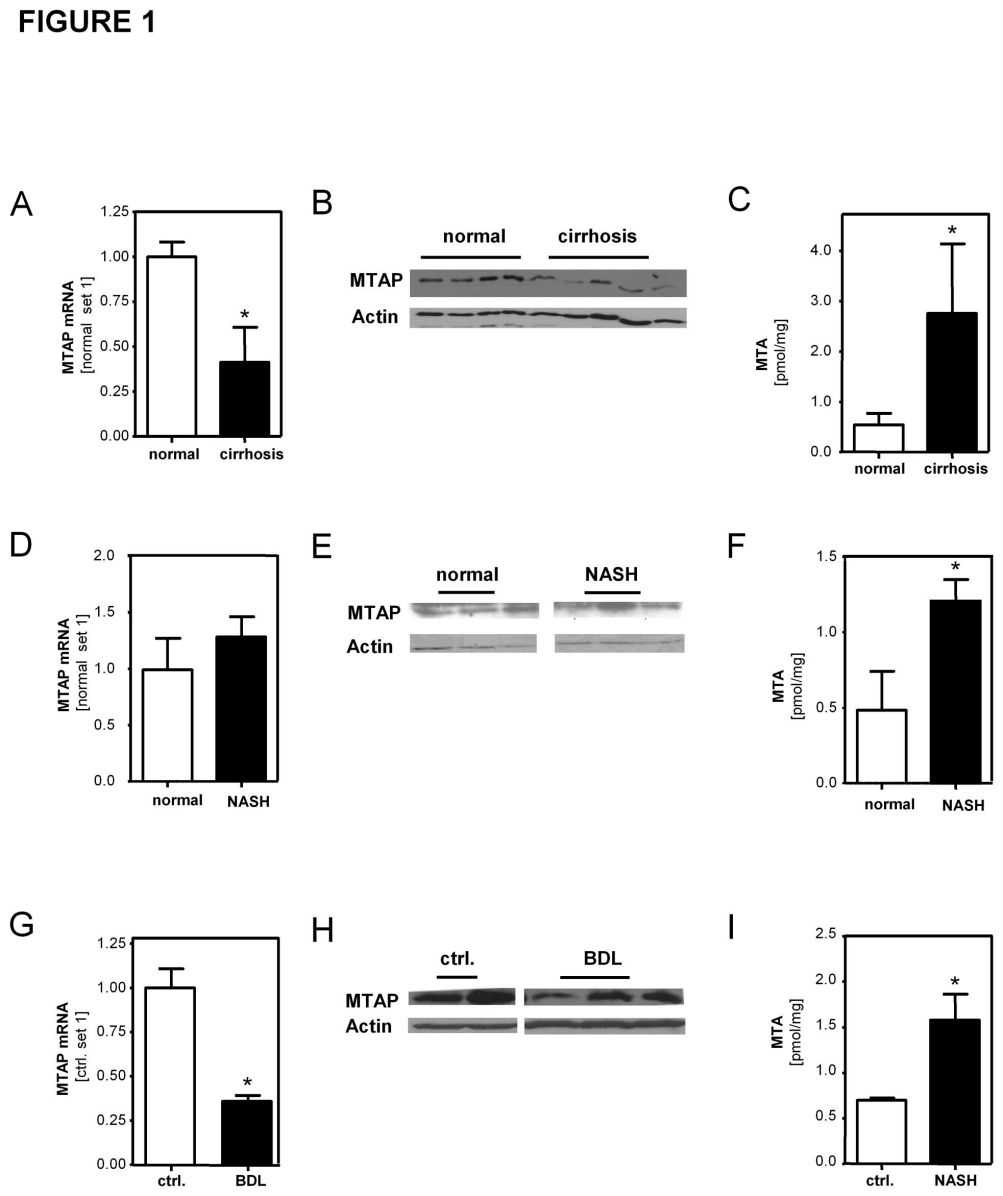
MTAP expression and hepatic MTA levels in chronic liver disease. Analysis of MTAP mRNA (**A**,**D**) and protein (**B**,**E**) expression in liver specimens obtained from patients with cirrhosis (n=8) and NAFLD (n=11), respectively, and controls without liver disease (n=6) by means of qRT-PCR and Western blotting. Hepatic MTA levels (**C**,**F**) in human liver tissues were determined by LC-ESI-MS/MS. (**G**,**H**) MTAP expression in BDL-treated (n=5) and sham operated control mice (n=4). (**I**) Hepatic MTA levels in a dietary murine NASH-model (n=5) compared to mice fed a control diet (n=5). In Western blot analysis, actin served as loading control. (*p<0.05).

First, we analyzed hepatic tissue from patients with alcoholic liver disease and chronic viral infection and found significant lower MTAP mRNA and protein expression in liver cirrhosis compared to normal liver tissue (Figure **1A and 1B**). In line with the downregulation of MTAP, hepatic levels of MTA were significantly higher in cirrhotic compared to normal human liver tissue (Figure **1C**). Next, we assessed patients with non-alcoholic steatohepatitis (NASH), in which fibrosis was less advanced. Expression of MTAP mRNA and protein were similar to that in normal hepatic tissue (Figure **1D and 1E**). Nevertheless, levels of MTA were significantly higher in NASH (Figure **1F**). To verify these findings in experimental models of chronic hepatic injury, we analyzed MTAP expression in mice subjected to three weeks of bile duct ligation (BDL) or fed with a NASH-inducing diet for 30 weeks. Similar as in human cirrhosis, MTAP mRNA and protein expression were significantly reduced in the BDL model (Figure **1G and 1H**). In murine NASH-livers, in concordance with the above human data, hepatic levels of MTA were significantly elevated (Figure **1I**) despite unaffected MTAP expression (Figure **S1**).

### MTAP expression in hepatocytes and hepatic stellate cells

**Figure 2:**
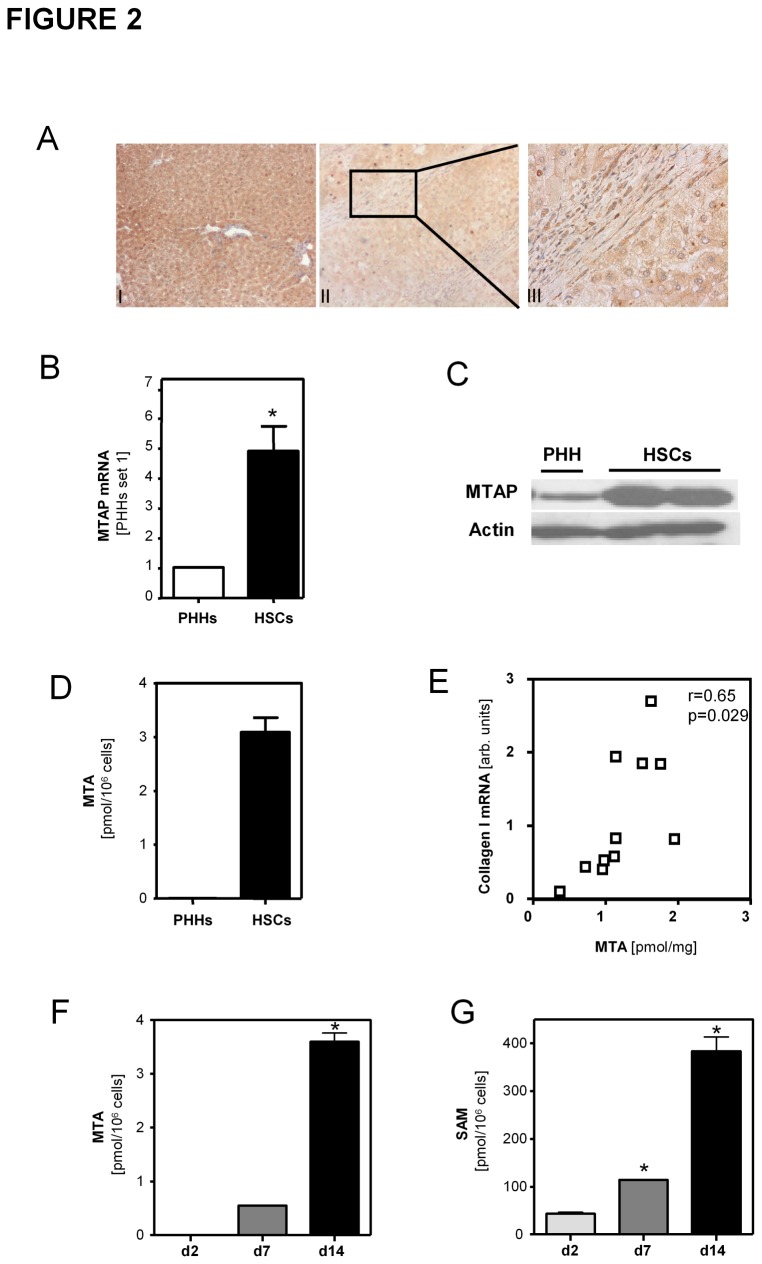
MTAP expression and MTA levels in hepatocytes and hepatic stellate cells. (**A**) Immunohistochemical analysis of MTAP in (**I**) control and (II,III) cirrhotic liver tissue (Magnification 200X and 400X). Measurement of MTAP mRNA (**B**) and protein (**C**) levels in primary human hepatocytes (PHH) and activated HSCs (*p<0.05 compared to PHH). In Western blot analysis actin staining was used to demonstrate equal protein loading. (**D**) Intracellular MTA levels measured by LC-ESI-MS/MS in PHHs compared to activated HSCs. (**E**) Correlation of hepatic MTA levels of NAFLD patients with hepatic collagen I mRNA expression. Intracellular (**F**) MTA and (**G**) S-adenosyl-L-methionine (SAM) levels in HSCs at different time points (days 2, 7, and 14) during the course of *in* *vitro* activation measured with LC-ESI-MS/MS. (*p<0.05 compared to d2) .

Immunohistochemical analysis revealed reduced MTAP expression in hepatocytes in both human cirrhotic liver tissue (Figure **2A**) and murine BDL-livers (Figure **S2**). In contrast, myofibroblast-like cells in fibrotic septa showed a strong MTAP immunosignal (Figure **2A**). Coimmunofluorescent staining confirmed that MTAP-expressing cells in fibrotic septa were also positive for alpha-smooth-muscle actin (α-sma), a specific marker for myofibroblasts such as activated hepatic stellate cells (HSCs) (Figure **S3**). Also *in vitro* activated HSCs revealed higher MTAP expression than primary human hepatocytes (PHHs) (Figure **2B,C**). Still, hepatocytes constitute the bulk mass of hepatic cells in healthy as well as in diseased livers, and thus, reduced MTAP expression in these cells likely accounts for reduced MTAP levels observed in fibrotic liver tissues. Furthermore, lowered MTAP in hepatocytes appeared as one probable reason for increased MTA levels in diseased livers. However, MTA levels were strikingly higher in activated HSCs compared to PHHs *in vitro* (Figure **2D**), and MTA levels correlated significantly with collagen I (Figure **2E**) and α-sma (data not shown) mRNA expression in diseased human liver tissue. Together these findings indicated that also activated HSCs contribute to elevated MTA levels in diseased livers. Higher levels of both MTAP expression and MTA in activated HSCs compared to hepatocytes might be explained by a generally more active methionine and adenine salvage pathway in response to high proliferation and cellular transdifferentiation in activated HSCs. In line with this, MTA levels were below the detection limit in freshly isolated HSCs and gradually increased during *in vitro* activation of HSCs (Figure **2F**). Also cellular levels of S-adenosyl-L-methionine (SAM), a key product of the methionine metabolism, progressively increased during the HSC activation *in vitro* (Figure **2G**). Similarly and in line with MTA, also SAM-levels in human NASH and cirrhotic liver tissues were significantly higher than in normal liver tissue (Figure **S4**). Together, these findings may also explain the observed increase in MTA levels in murine and human NASH tissues despite unaltered MTAP expression (Figure 1D-F).

### Functional role of MTAP in hepatic stellate cells

**Figure 3:**
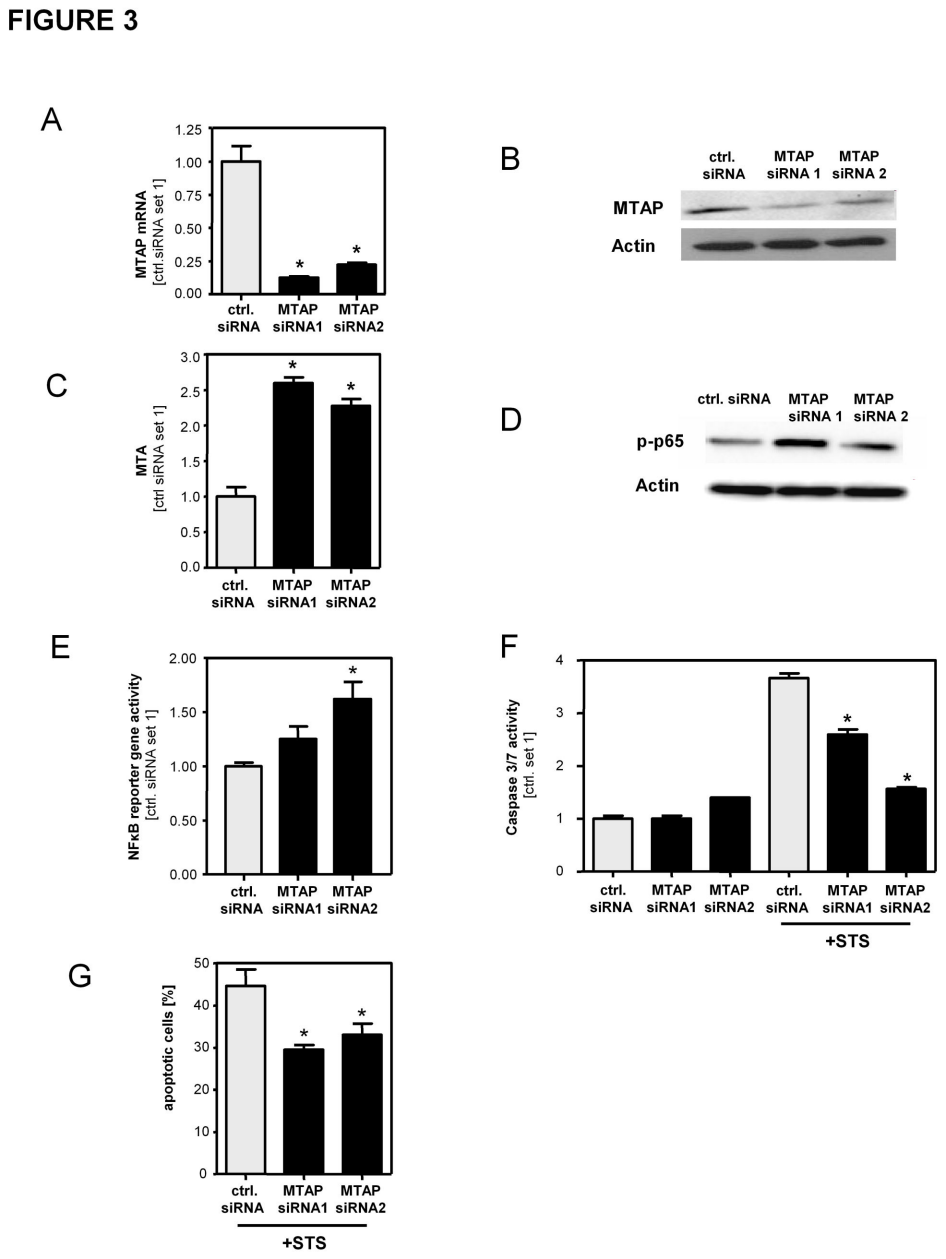
Suppression of MTAP in activated hepatic stellate cells with siRNA. Activated HSCs were transfected with siRNA against MTAP (siRNA1 and siRNA2) or control siRNA. (**A**,**B**) Analysis of MTAP expression by qRT-PCR and Western blotting. (**C**) Quantification of cellular MTA levels by means of LC-ESI-MS/MS. (**D**) Western blot analysis of phosphorylated p65. Actin staining was used to demonstrate equal protein loading. (**E**) NFκB reporter gene assay. (**F**) Analysis of caspase3/7 activity in HSCs with and without staurosporine (STS) treatment (500nM; 4h) to induce apoptosis. (**G**) Assessment of STS-induced apoptosis by flow cytometric analysis of annexin V-FITC / propidium iodide stained cells. Depicted are mean percentages of total apoptotic cells from 3 independent experiments. (*p<0.05 compared to ctrl. siRNA).

To gain insight into the functional role of MTAP in HSCs, we transiently transfected activated HSCs with siRNA directed against MTAP. This led to significantly reduced MTAP mRNA and protein expression (Figure **3A,B**; Figure **S5**) compared to cells transfected with control siRNA. Furthermore, MTAP suppression led to increased MTA levels in HSCs (Figure **3C**). Previously, we had shown that MTA affected NFκB activity in hepatocellular carcinoma [7]. Here, we assessed whether MTA affected this signaling pathway also in HSCs. MTAP-suppressed HSCs revealed higher levels of phosphorylated p65 (Figure **3D**) and a NFκB reporter assay showed increased NFκB activity in MTAP suppressed cells (Figure **3E**). The NFκB signaling pathway is increased during the activation of HSCs and protects these cells from apoptosis [11,24]. Consequently, analysis of caspase3/7 activity (Figure **3F**) and FACS analysis (Figure **3G**) revealed that HSCs with suppressed MTAP expression were less susceptible to staurosporine (STS) induced apoptosis compared to control cells. 

**Figure 4:**
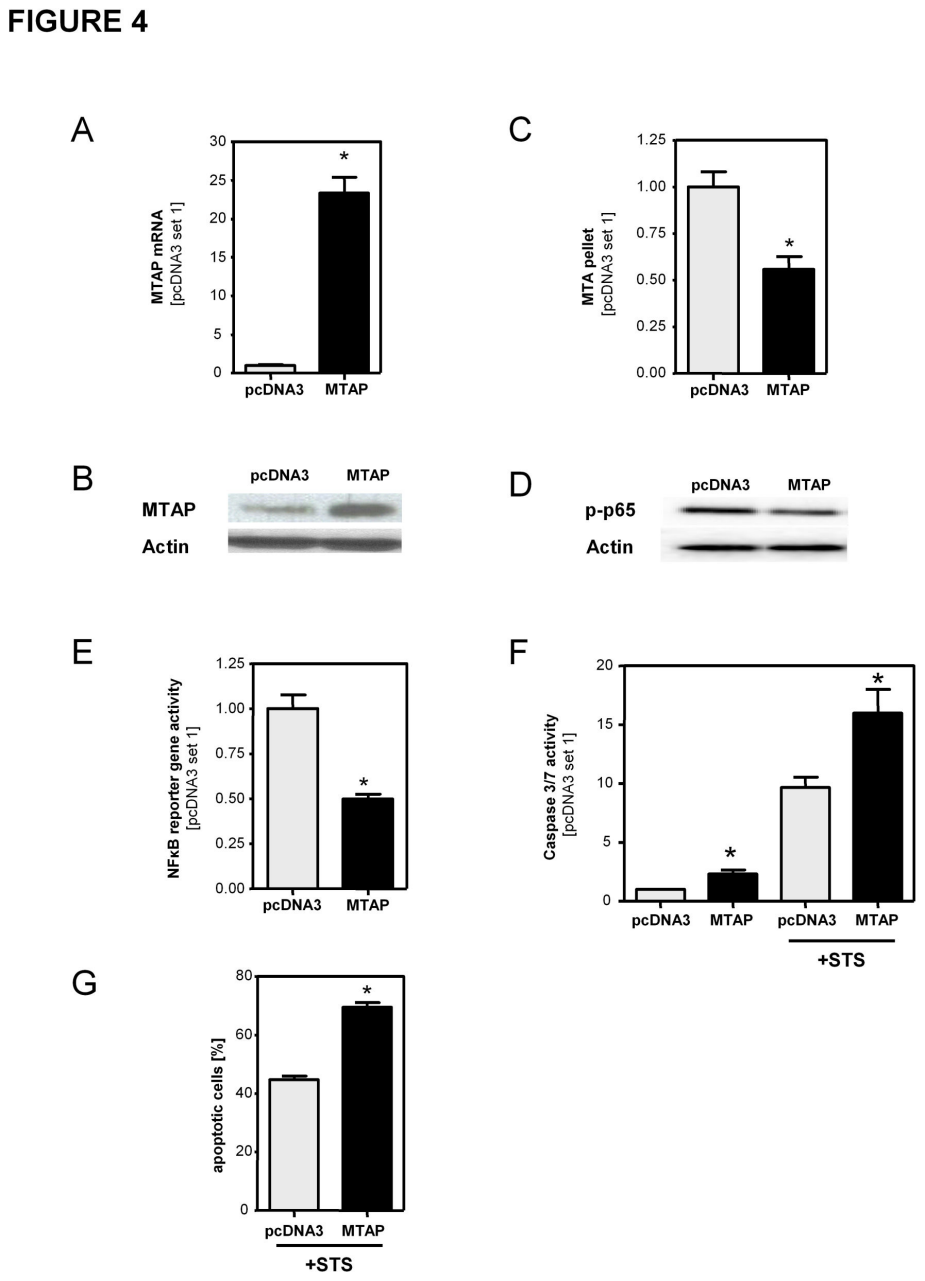
Overexpression of MTAP in activated HSCs. Activated HSCs were transiently transfected with control vector (pcDNA3) and an MTAP expression vector (MTAP). (**A**,**B**) Analysis of MTAP expression by qRT-PCR and Western blotting. (**C**) Quantification of cellular MTA levels by LC-ESI-MS/MS. (**D**) Western blot analysis of phosphorylated p65. Actin staining was used to demonstrate equal protein loading. (**E**) NFκB reporter gene assay. (**F**) Analysis of caspase3/7 activity in HSCs with and without staurosporine (STS) treatment (500nM; 4h) to induce apoptosis. (**G**) Assessment of STS-induced apoptosis by flow cytometry applying annexin V and propidium iodide staining. Depicted are mean percentages of total apoptotic cells from 3 independent experiments. (*p<0.05 compared to pcDNA3).

In a second approach, we enhanced MTAP expression in activated HSCs by transient transfection with a MTAP-expression plasmid (Figure **4A,B**). Consequently, MTAP overexpressing HSCs revealed reduced MTA levels (Figure **4C**) and reduced p65-phosphorylation (Figure **4D**) and NFκB reporter gene activity (Figure **4E**). Furthermore, elevated MTAP expression made HSCs more susceptible to STS induced apoptosis (Figure **4F,G**) than control vector (pcDNA3) transfected cells. 

Together, these data indicated that MTAP expression was a critical regulator of MTA levels in activated HSCs, and herewith significantly affected NFκB activity and apoptosis in HSCs.

### Effect of MTA on hepatic stellate cells

**Figure 5:**
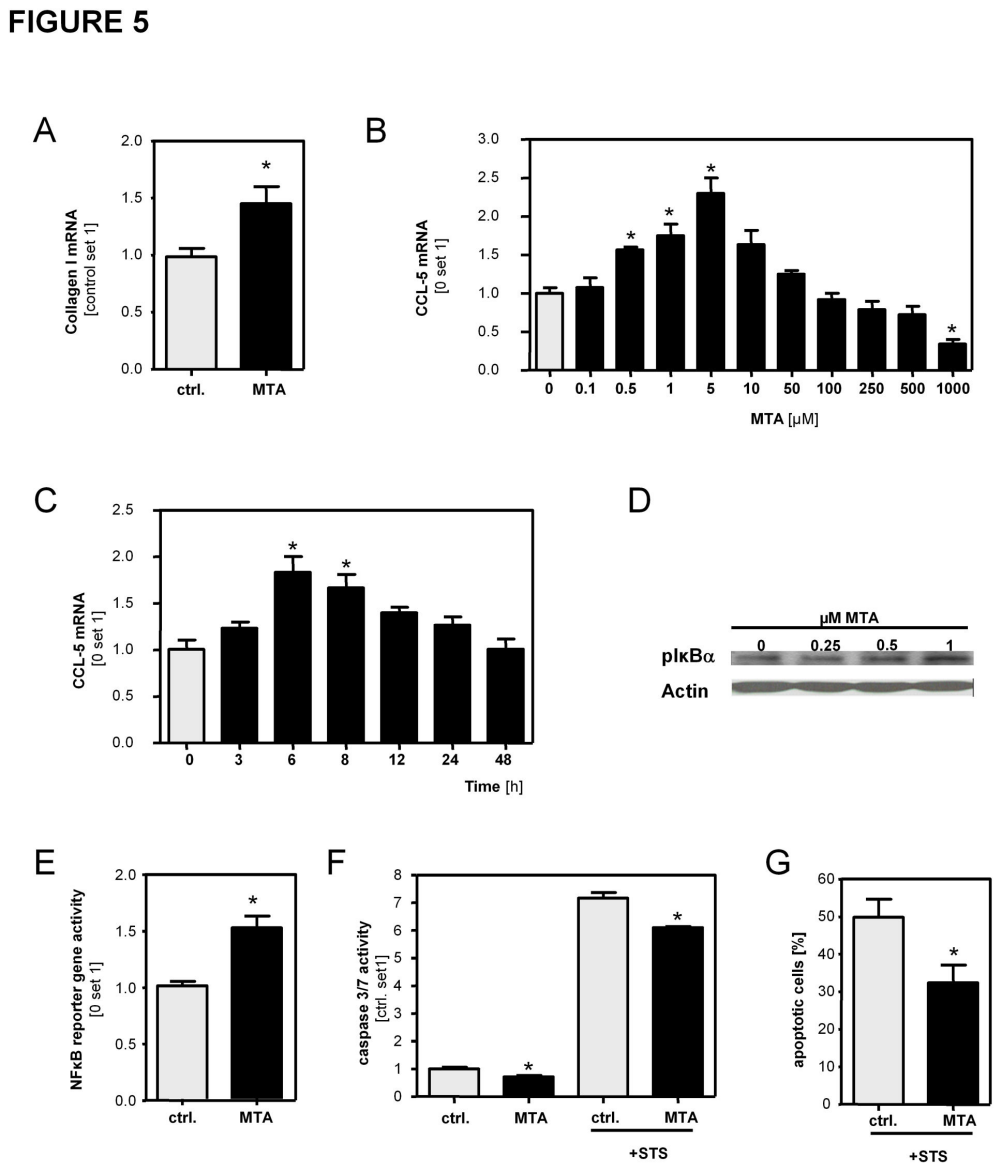
Effect of MTA stimulation on hepatic stellate cells. (**A**) Effect of MTA on hepatic stellate cell activation *in* *vitro*. Two days after isolation HSC were incubated with MTA (1µm) for 72h. Subsequently, collagen I mRNA expression was analyzed by quantitative PCR. (**B**,**C**) CCL5 mRNA expression in activated HSCs treated with MTA at different doses and for different time intervals. (**D**) Analysis of phosphorylated IκBα by Western Blot analysis and (**E**) NFκB reporter gene assay in activated HSCs stimulated with MTA (1µM). (**F**) Analysis of caspase3/7 activity in MTA (1µM) treated HSCs with and without staurosporine (STS) treatment (500 nM; 4h) to induce apoptosis. (**G**) Assessment of STS-induced apoptosis in MTA (1µM) treated and control HSCs by annexin V-FITC / propidium iodide staining and flow cytometry. Depicted are mean percentages of total apoptotic cells from 3 independent experiments. (*p<0.05 compared to control).

Loss and gain of function studies indicated that MTAP regulated MTA levels exhibit profibrogenic effects on HSCs. Moreover, we observed that stimulation with 1µM MTA, i.e. a MTA concentration found in diseased murine and human liver tissues, induced the activation of HSCs *in vitro* as indicated by increased collagen type I and α-sma expression (Figure **5A** and Figure **S6**). In contrast, previous studies by Simile et al. and Latasa et al. showed that MTA stimulation in a dose range between 25µM and 500µM or 200µM and 500µM, respectively, caused impaired fibrogenic characteristics of activated HSCs [25,26]. To unravel these putative discrepancies we stimulated activated HSCs with MTA in a wide dose range comprising MTA levels found in diseased liver tissues (up to 5µM) and MTA doses as high as 1mM. In concentrations up to 5µM MTA stimulation led to dose-dependent induction of CCL5 (also called RANTES (Regulated on Activation, Normal T cell Expressed and Secreted)) and CCL2 (also referred to as monocyte chemotactic protein-1 (MCP-1)) expression, while higher MTA doses reduced the expression of both chemokines in activated HSCs (Figure **5B** and Figure **S7A**). Similarly, expression of collagen type I and transforming growth factor beta (TGF-β) was induced by lower MTA doses but reduced in response to higher MTA doses (Figure **S7B,C**). These findings indicated a nonmonotonic dose-response relationship between MTA levels and proinflammatory and profibrogenic gene expression in activated HSCs. While the studies of Simile et al. and Latasa et al. [25,26] comprehensively assessed the effects of exogenously applied MTA doses, we subsequently focused on the assessment of the effects of (endogenous) MTA levels found in diseased livers. In this dose range, MTA caused a time-dependent induction of CCL5 (Figure **5C**) and MCP-1 (data not shown) expression in activated HSCs *in vitro*. Rapid induction of chemokine expression pointed to an effect on transcriptional regulation, and NFκB is a known critical regulator of these chemokines [27]. MTA stimulation of HSCs induced a dose-dependent phosphorylation of IκB-α (Figure **5D**) and activation of NFκB-reporter gene activity (Figure **5E**). Moreover, analysis of caspase3/7 activity and FACS analysis revealed, that MTA stimulation increased the resistance of activated HSCs towards apoptosis (Figure **5F,G**). 

### Functional effect of MTAP/MTA on survivin expression in activated hepatic stellate cells

**Figure 6:**
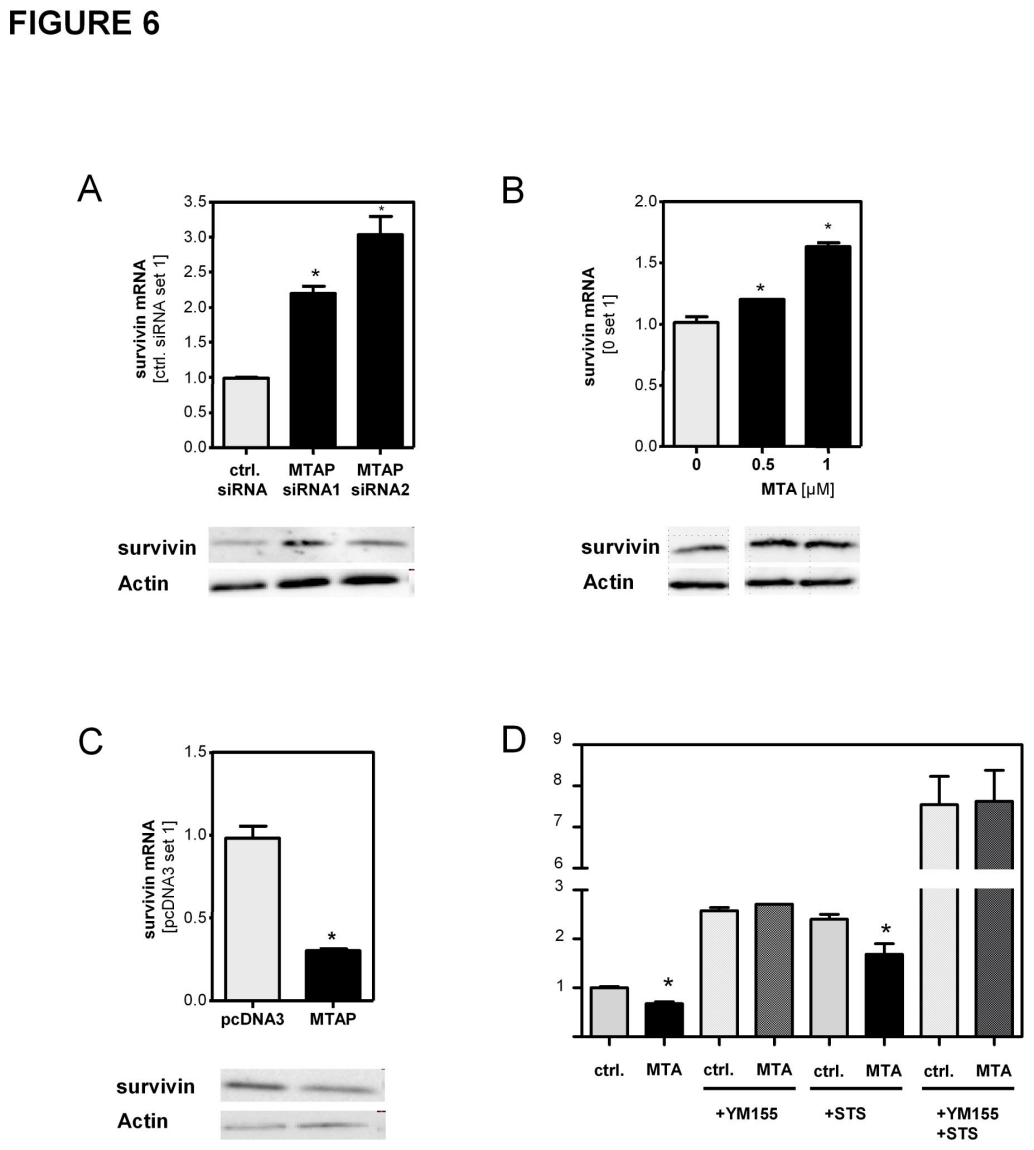
Functional effect of MTAP/MTA on survivin expression in activated hepatic stellate cells. Survivin mRNA and protein expression in activated HSCs (**A**) transfected with MTAP siRNA or control siRNA, (**B**) stimulated with MTA, and (**C**) transfected with a MTAP expression plasmid or empty vector (pcDNA3); (*p<0.05 compared to ctrl.; ctrl. siRNA; 0µM MTA or pcDNA3, respectively). (**D**) Assessment of caspase3/7 activity in MTA-treated HSCs with and without preincubation with the survivin inhibitor YM155 (5µM) and with and without staurosporine (STS 500 nM; 4h) treatment to induce apoptosis. (*p<0.05 compared to ctrl.).

Manipulation of MTAP expression or MTA stimulation did not significantly affect BAX, BCLXL and XIAP expression in activated HSCs (Figure **S8**). However, suppression of MTAP expression (Figure **6A**) and wild-type HSCs stimulated with MTA (Figure **6B**) revealed an increased expression of survivin. In contrast, survivin expression was downregulated in MTAP overexpressing HSCs compared to pcDNA3 transfected control cells (Figure **6C**). Survivin is a target of NFκB [28] and can prevent caspase-induced apoptosis [29]. This suggested that the regulation of this anti-apoptotic factor accounted at least in part for the effects of the manipulation of MTAP levels and MTA stimulation, respectively, on apoptosis resistance of HSCs. Accordingly, pretreatment with the survivin inhibitor YM-155 abolished the apoptosis protecting effect of MTA stimulation in HSCs (Figure **6D**). In summary, these findings indicated, that NFκB-mediated regulation of survivin expression was responsible for the effects of MTAP and MTA on apoptosis resistance of activated HSCs.

### Regulation of MTAP expression in activated hepatic stellate cells

**Figure 7:**
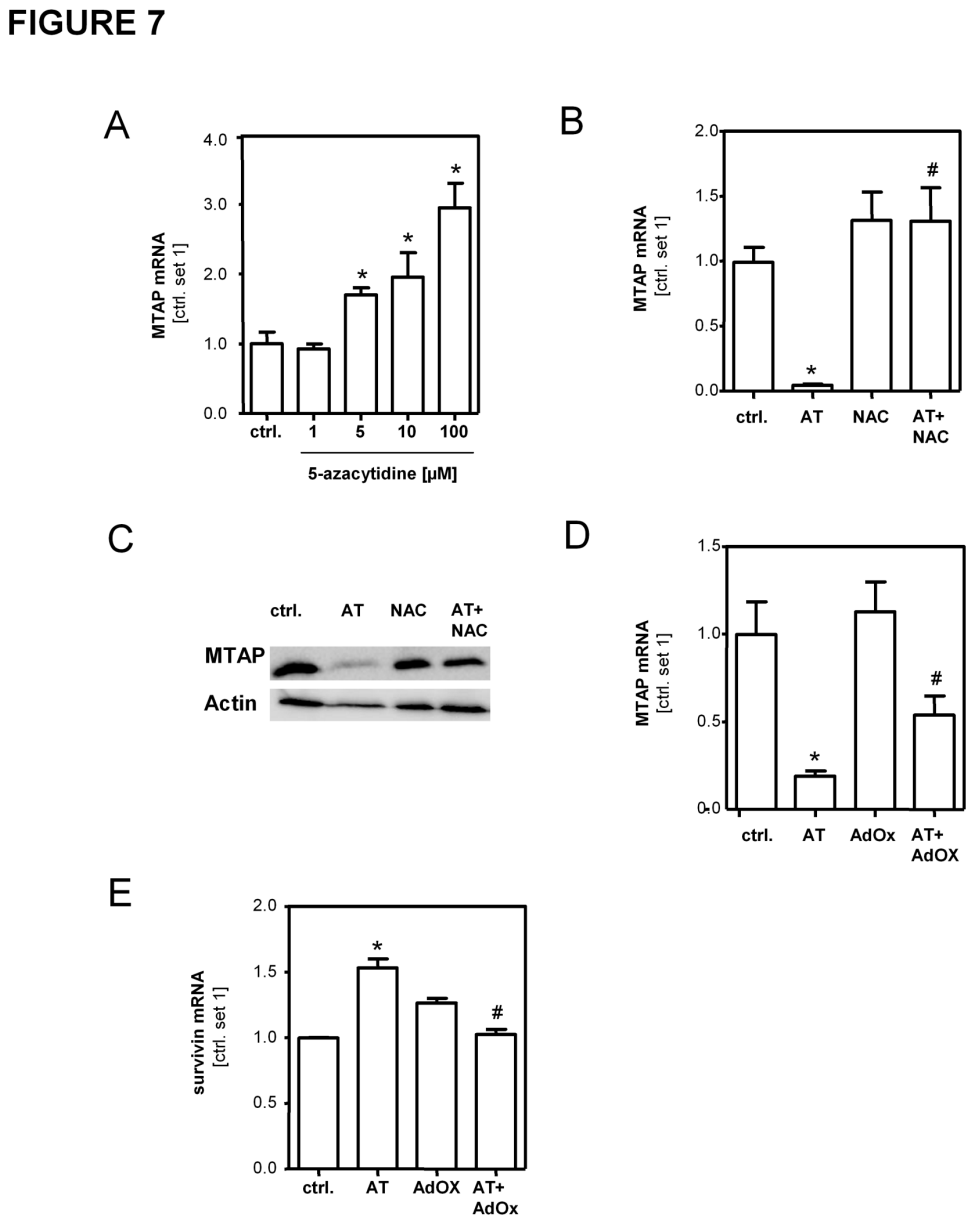
Regulation of MTAP expression in activated hepatic stellate cells. MTAP expression in activated HSCs treated with 5-azacytidine (1-100µM) (**A**) or As_2_O_3_ (AT) (10µM) and NAC (10mM) (**B**,**C**). MTAP and survivin mRNA expression in HSCs stimulated with methylation inhibitor AdOx (50µM) alone and in combination with AT (10µM) (**D**,**E**). (*p<0.05 compared to ctrl.; ^#^p<0.05 compared to AT).

Next, we analyzed the mechanisms that regulate MTAP expression in activated HSCs. We had shown previously, that promoter methylation caused downregulation of MTAP expression in HCC cells [30]. To assess whether this mechanism also affected MTAP expression in HSCs, we incubated activated HSCs with the demethylating agent 5-azacytidine (5-Aza) and found that this treatment enhanced MTAP expression in a dose dependent manner (Figure **7A**). Liver damage is characterized by increased formation of reactive oxygen species (ROS), and oxidative stress has been shown to induce promoter methylation [31,32]. Arsenic trioxide (AT) induces ROS production *via* up-regulation of NADPH oxidase [33] (Figure **S9**), and AT treatment inhibited MTAP mRNA and protein expression (Figure **7B,C**). Preincubation with the ROS-scavenger N-Acetyl-L-Cysteine (NAC) abrogated AT-induced downregulation of MTAP mRNA and protein (Figure **7B,C**). Further, stimulation with hydrogen peroxide dose dependently downregulated MTAP in HSCs (Figure **S10**) confirming that oxidative stress caused a downregulation of MTAP in HSCs. In contrast, pretreatment with methyltransferase inhibitor adenosine periodate oxidized (AdOx) abolished ROS induced MTAP downregulation (Figure **7D**). On the contrary, AT stimulation increased survivin expression in activated HSCs, and this induction was blunted by AdOx treatment (Figure **7E**). Together, these data indicate that epigenetic mechanisms account at least in part for the increased MTAP expression while oxidative stress causes a downregulation of MTAP expression in activated HSCs, and herewith functionally affect the profibrogenic phenotype of these cells.

### Regulation of MTAP expression and MTA effects in hepatocytes

**Figure 8:**
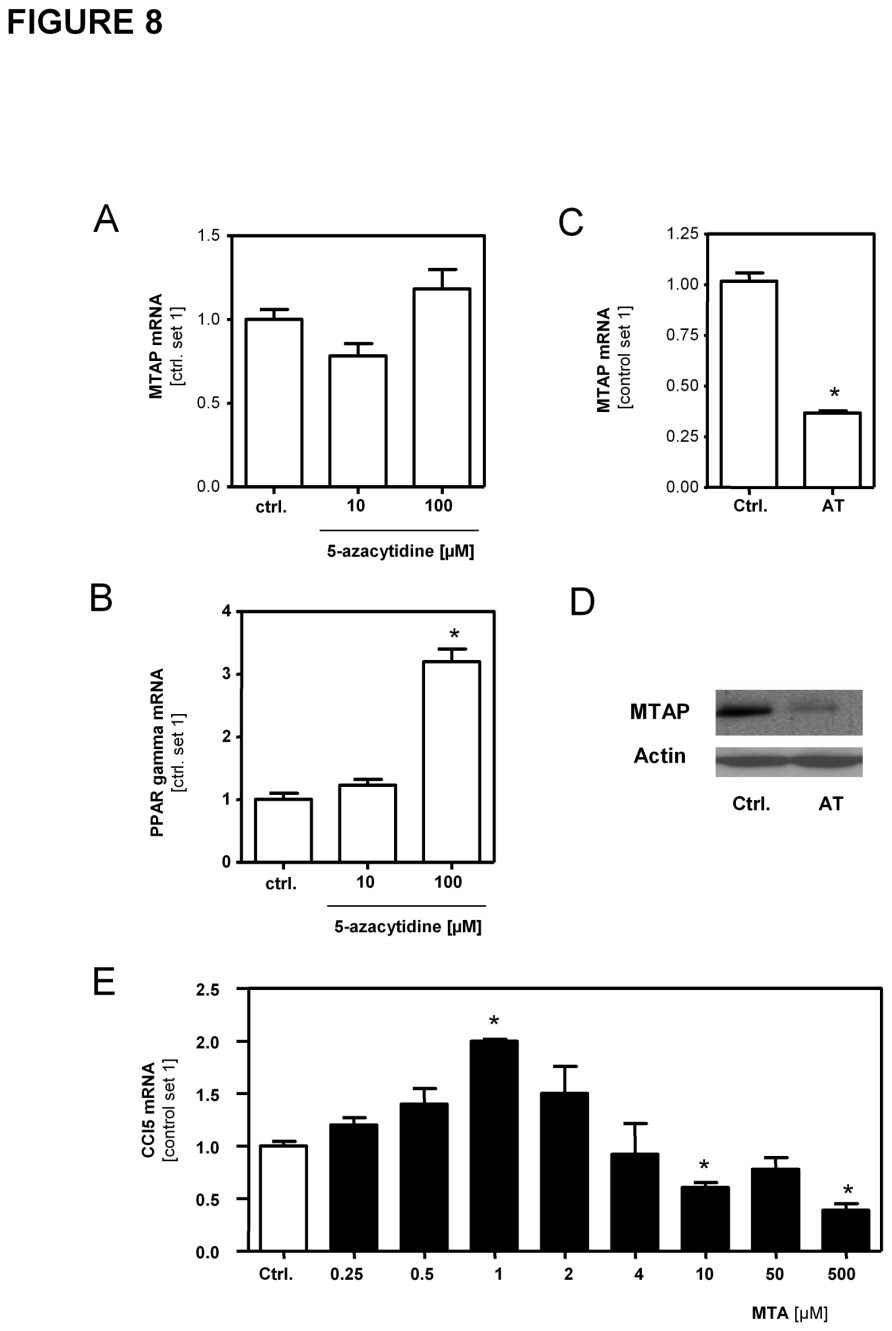
Regulation of MTAP expression and MTA effects on hepatocytes. (**A**,**B**) MTAP and PPAR-gamma mRNA expression in murine hepatocytes treated with 5-azacytidine (10µM and 100µM). (**C**,**D**) MTAP mRNA and protein expression in murine hepatocytes treated with As_2_O_3_ (AT) (10µM). (**E**) CCL5 mRNA expression in murine hepatocytes stimulated with different doses of MTA. (*p<0.05 compared to ctrl.).

Next, we wanted to analyze whether the molecular mechanisms identified to regulate MTAP expression in HSCs are also responsible for the observed downregulation of MTAP expression in hepatocytes in cirrhotic liver tissues (Figure 2A and Figure S2). In contrast to HSCs (Figure 7A), treatment with the demethylating agent 5-Aza did not significantly affect MTAP expression in primary murine and human hepatocytes (Figure **8A** and Figure **S11**). However, expression of proliferator-activated receptor-gamma (PPAR-gamma), a gene known to be regulated by promoter-methylation [34,35], significantly increased in response to 5-Aza treatment (Figure **8B**). To assess the role of oxidative stress in MTAP regulation in hepatocytes, cells were incubated with arsenic trioxide. This inducer of ROS production significantly reduced MTAP expression in primary hepatocytes (Figure **8C,D**). These findings indicate, that oxidative stress, a known inducer of hepatocellular inflammation and fibrosis [36,37], significantly impairs MTAP expression in both activated HSCs and hepatocytes, and herewith, also greatly affects hepatic MTA levels. To analyze whether MTA also exhibits similar pro-inflammatory effects on hepatocytes as observed in HSCs, primary murine hepatocytes were stimulated with MTA in a wide dose-range comprising MTA levels found in diseased liver tissues and pharmacological doses MTA doses as high as 500µM. In concentrations up to 5µM MTA stimulation led to induction of CCL5 expression, while higher MTA doses reduced the expression of this chemokine in primary murine hepatocytes (Figure **8E**). Similarly as in HSCs, these findings indicate a nonmonotonic dose-response relationship between MTA levels and proinflammatory gene expression also in hepatocytes.

## Discussion

Recently, we have shown downregulation and tumor suppressor activity of MTAP in hepatocellular carcinoma (HCC) [7]. Here, we expanded our investigation on chronic liver disease, which is the major HCC precondition. The observed downregulation of MTAP mRNA expression in cirrhotic human livers and experimental models of liver cirrhosis was in line with a previous study by Berasain et al. [8]. Here, we confirmed this finding at the protein level, and applying immunohistochemistry we revealed that hepatocytes of cirrhotic livers have lower MTAP expression than hepatocytes in normal liver tissue. In contrast, we found strong MTAP expression in activated HSCs in cirrhotic livers. Moreover, we demonstrated that not only MTAP but also its metabolite MTA are abundant in activated HSCs. This may be caused by an upregulation of polyamine biosynthesis in response to enhanced proliferation and cellular transdifferentiation during the course of HSC activation. In line with this, we found increased MTA as well as SAM levels during *in vitro* activation of HSCs. Interestingly, fully activated HSCs showed markedly higher MTA levels than hepatocytes *in vitro*. Moreover, hepatic MTA levels strongly correlated with collagen I expression in diseased hepatic tissue. Together these findings indicated that also *in vivo* activated HSCs significantly contribute to MTA abundance in fibrotic livers despite their relatively lower quantity compared to hepatocytes, which are the main cellular source of total MTA in normal liver tissue. Still, our data cannot define the exact contribution of activated HSCs and hepatocytes to MTAP and MTA levels in diseased livers. Likely the contribution also varies during the course of chronic liver disease and depending on the type of liver injury. Thus, differences in the cellular sources of MTAP and MTA could also account for enhanced MTA levels in NASH, although MTAP levels in total liver tissue were similar as in normal liver tissue. Furthermore, this may be an explanation why Berasain et al. did not find MTA accumulation in the model of carbon tetrachloride (CCl_4_)-induced liver injury in spite of a compromised expression of MTAP [8]. In addition, it has to be considered that MTA is produced during polyamine biosynthesis from S-adenosylmethionine (SAM), its metabolic precursor. Interestingly, Berasain et al. found that SAM was strongly reduced in CCl_4_-treated rats, and herewith, identified one further potential mechanism why reduced MTAP levels in diseased livers may not always coincide with enhanced MTA levels in particular experimental models.

In the present study, we observed increased MTA levels in two experimental models of hepatic injury as well as in patients with NASH and cirrhosis of different origin. Stimulation of activated HSCs with MTA at concentrations similar to those found in diseased liver tissues caused increased profibrogenic gene expression and NFκB activation, as well as enhanced apoptosis resistance. In contrast to our findings, some groups have reported proapoptotic effects of MTA on hepatoma cells [38], and found antifibrotic effects of MTA on HSCs *in vitro* and in experimental models of hepatic fibrosis [25,26]. However, in those studies significantly higher, pharmacological doses had been administered, whereas the MTA levels achieved here mirrored endogenous hepatic levels. Moreover, pharmacological doses of MTA have been shown to exhibit protective effects on hepatocytes *in vitro* [38–40], while MTA accumulation in HCC promotes tumorigenicity [7]. Our *in vitro* findings clearly showed a biphasic effect of MTA stimulation levels on profibrogenic response in activated HSCs and hepatocytes, and it is reasonable that also in other cells such a nonmonotonic dose-response relationship exists. Together, these findings indicate that hepatic effects of MTA are a double-edged sword and warrant the exercise of caution in the pharmacological use of MTA in treating liver disease, as pharmacological levels may drop to levels at which profibrogenic and pro-tumorigenic effects predominate. 

In addition to exogenous MTA stimulation also manipulation of MTAP expression and subsequent alterations of intracellular MTA levels functionally affected activated HSCs. Survivin has been described as a NFκB target gene in tumor cells [28], and De Minicis et al. described increased survivin expression in HSCs isolated from murine fibrosis models [41]. Here, we showed that survivin functionally affected apoptosis resistance of activated HSCs and identified this member of the Inhibitor of Apoptosis (IAP) family as a transcriptional target of exogenous MTA mediated NFκB activation in activated HSCs. Moreover, loss and gain of function studies revealed that MTAP-regulated levels of intracellular MTA affect NFκB activity and survivin expression in activated HSCs. NFκB activity in HSCs is critical for their resistance against apoptosis, and herewith, the extent of fibrosis in chronic liver injury [12]. Hepatic fibrosis and cirrhosis are HCC preconditions, and we have shown downregulation and tumor suppressor activity of MTAP in HCC [7]. Together, these findings suggest (induction of) MTAP as therapeutic strategy against both fibrosis and cancerogenesis in chronic liver disease. Noteworthy, we found that promoter methylation is a critical regulator of MTAP expression in HSCs similar as we and others have shown before in HCC cells [8,30]. DNA methylation inhibitors have been suggested for treatment of HCC [42] and have been shown to inhibit HSC activation and fibrogenesis [43]. Our study unraveled a novel mechanism by which epigenetic mechanisms critically affect the fibrogenic potential of HSCs. Furthermore, we identified oxidative stress to impair MTAP expression in HSCs as well as in hepatocytes. This finding is complementary to a previous study by Fernandez-Irigoyen et al., who demonstrated redox regulation of MTAP activity in hepatocytes [44].

In conclusion, we identified regulation of MTAP expression and corresponding MTA levels as a novel mechanism affecting profibrogenic gene expression, NFκB activity and apoptosis resistance in HSCs. This may be exploited for the prognosis or treatment of fibrosis progression in chronic liver disease.

## Supporting Information

Figure S1 **MTAP expression in a dietary murine NASH model.**
(TIF)

Figure S2 **MTAP expression in murine fibrotic tissue.**
(TIF)

Figure S3 **MTAP expression in alpha-smooth muscle actin positive cells in fibrotic septa in cirrhotic livers.**
(TIF)

Figure S4 **Hepatic S-adenosyl-L-methionine in NASH and cirrhosis.**
(TIF)

Figure S5 **MTAP expression in activated HSCs.**
(TIF)

Figure S6 **Effect of MTA on hepatic stellate cell activation.**
(TIF)

Figure S7 **Effect of MTA stimulation on activated hepatic stellate cells.**
(TIF)

Figure S8 **Expression of apoptosis related genes in MTAP manipulated and MTA treated activated HSCs.**
(TIF)

Figure S9 **ROS induction and induction of p47phox by arsenic trioxide stimulation in activated HSCs.**
(TIF)

Figure S10 **Induction of ROS with hydrogen peroxide and MTAP expression in H_2_O_2_ treated HSCs.**
(TIF)

Figure S11 **Regulation of MTAP expression in hepatocytes.**
(TIF)
