# Supplementary figures and images for: Expression and Function of Methylthioadenosine Phosphorylase in Chronic Liver Disease
Source: PLoS One. 2013 Dec 6;8(12):e80703. doi: 10.1371/journal.pone.0080703 (PMC3855635; doi:10.1371/journal.pone.0080703)

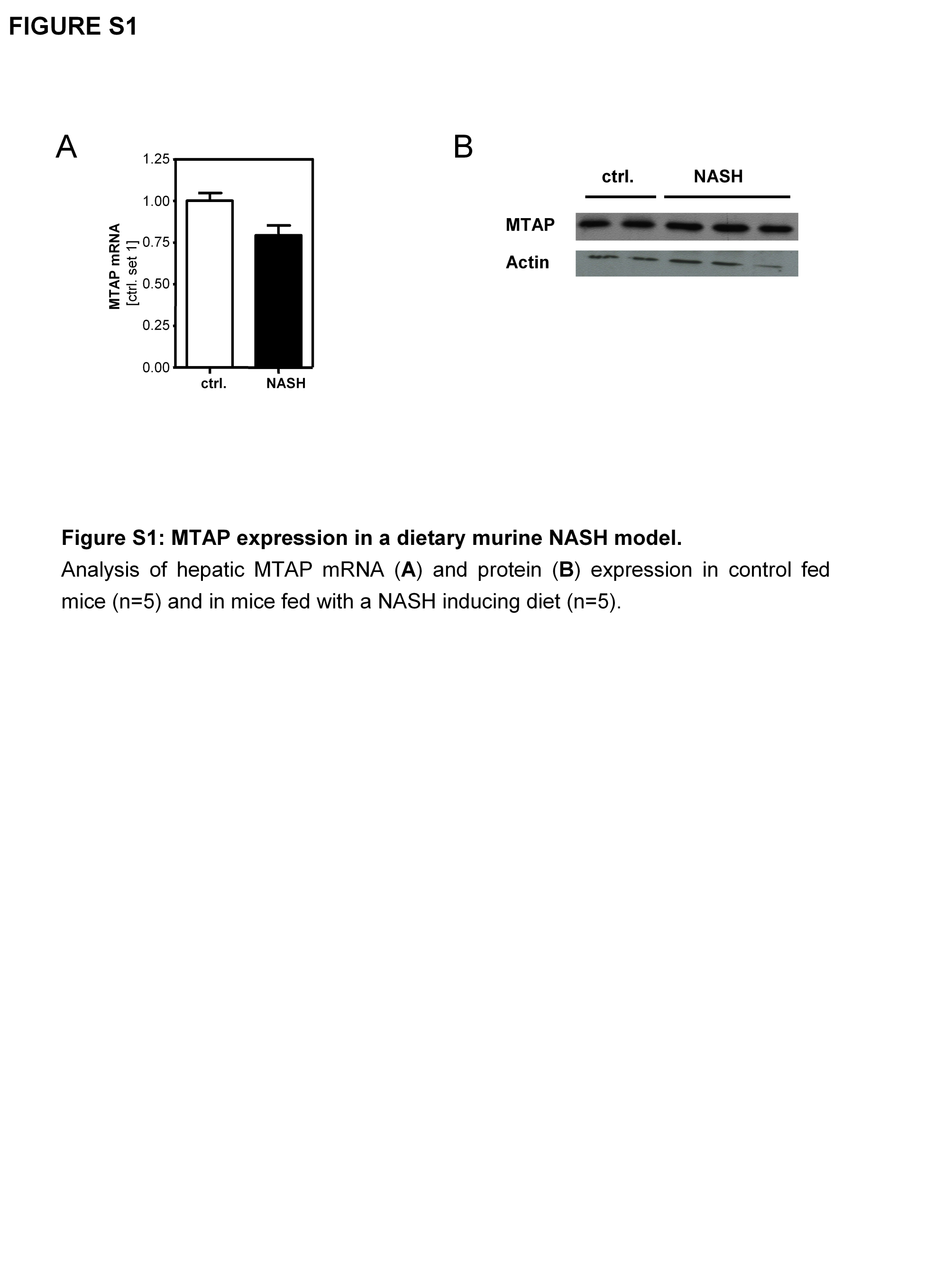

Supplement: Figure S1 — MTAP expression in a dietary murine NASH model. (TIF) [file pone.0080703.s001.tif]

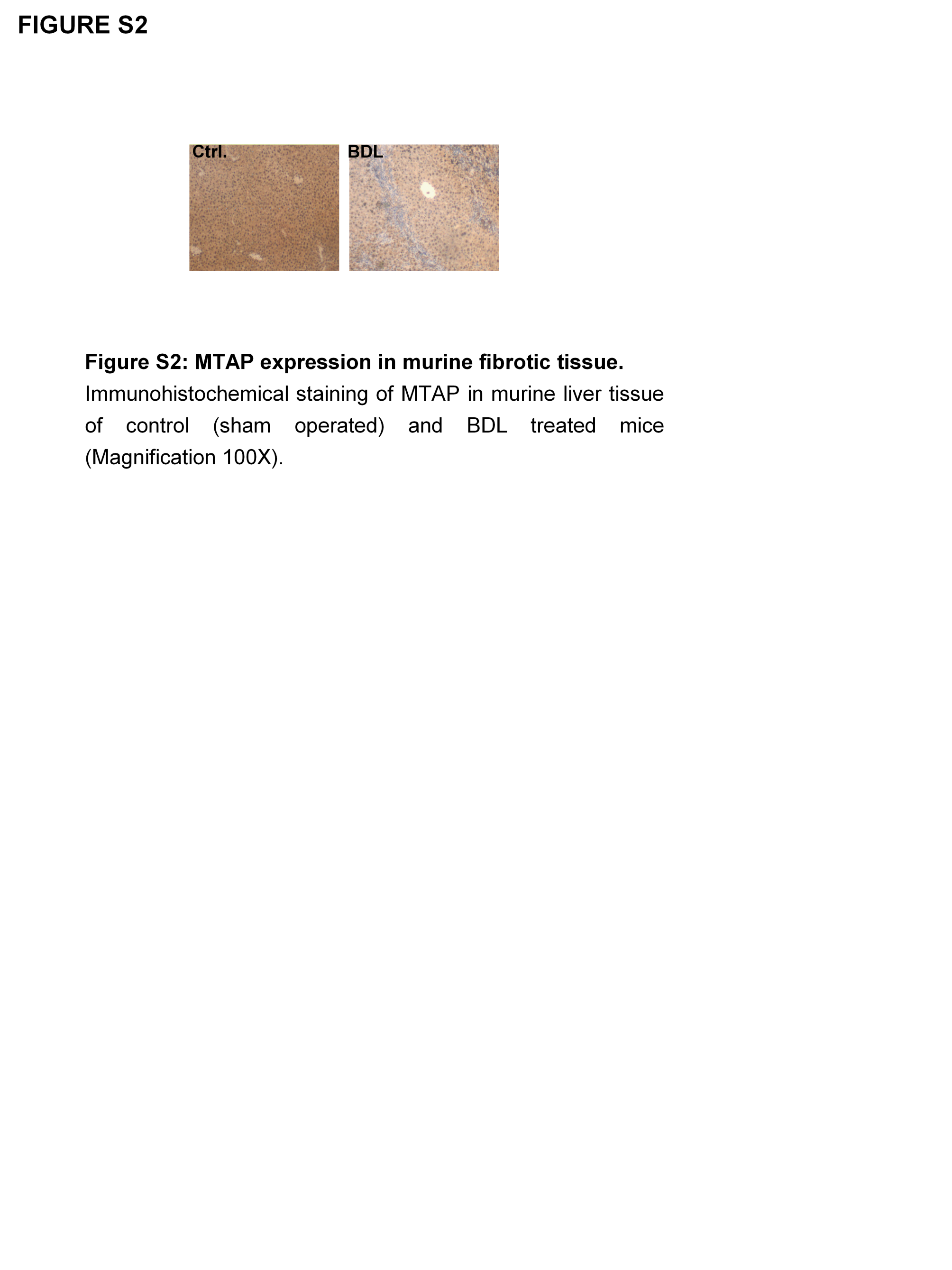

Supplement: Figure S2 — MTAP expression in murine fibrotic tissue. (TIF) [file pone.0080703.s002.tif]

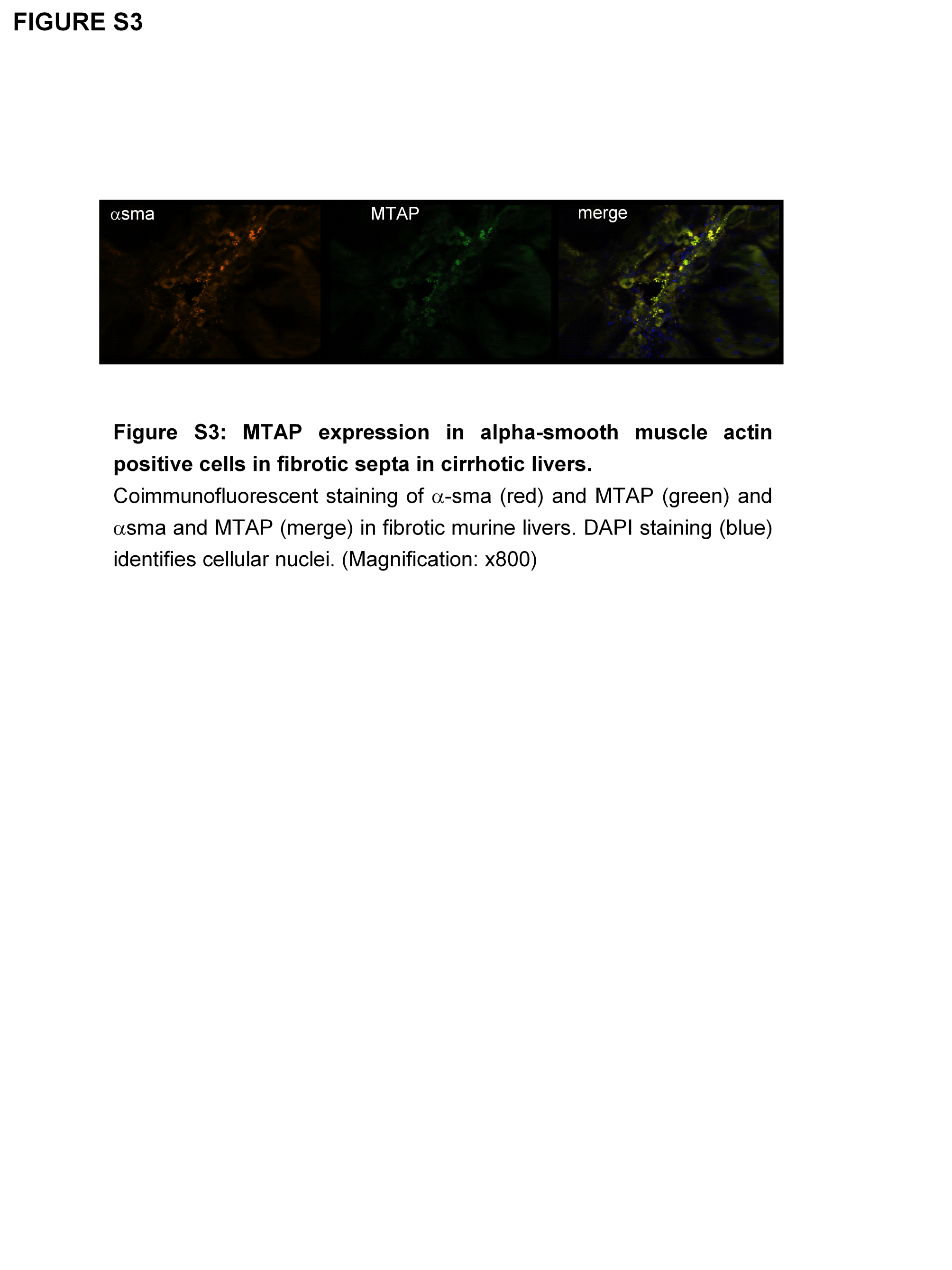

Supplement: Figure S3 — MTAP expression in alpha-smooth muscle actin positive cells in fibrotic septa in cirrhotic livers. (TIF) [file pone.0080703.s003.tif]

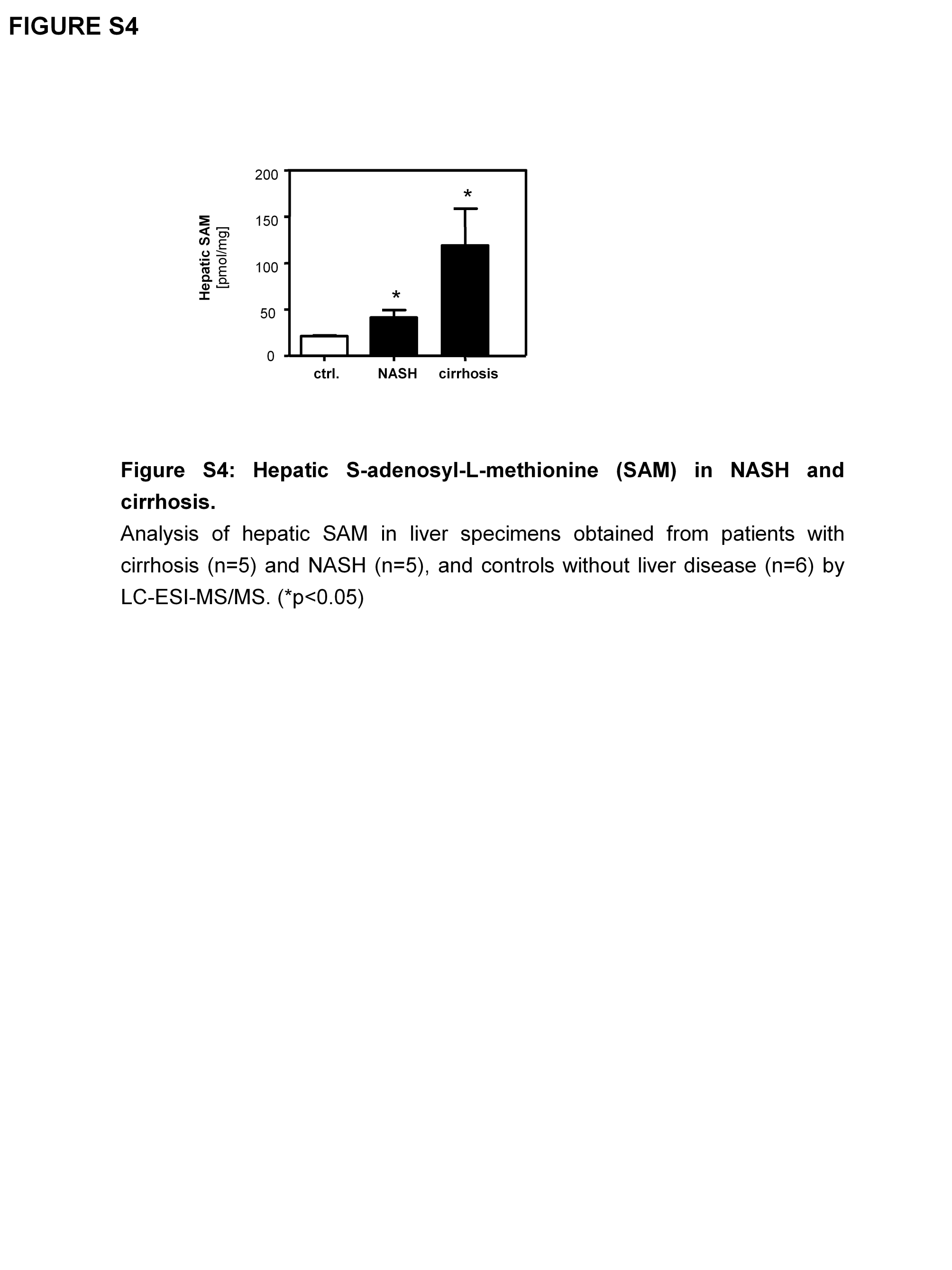

Supplement: Figure S4 — Hepatic S-adenosyl-L-methionine in NASH and cirrhosis. (TIF) [file pone.0080703.s004.tif]

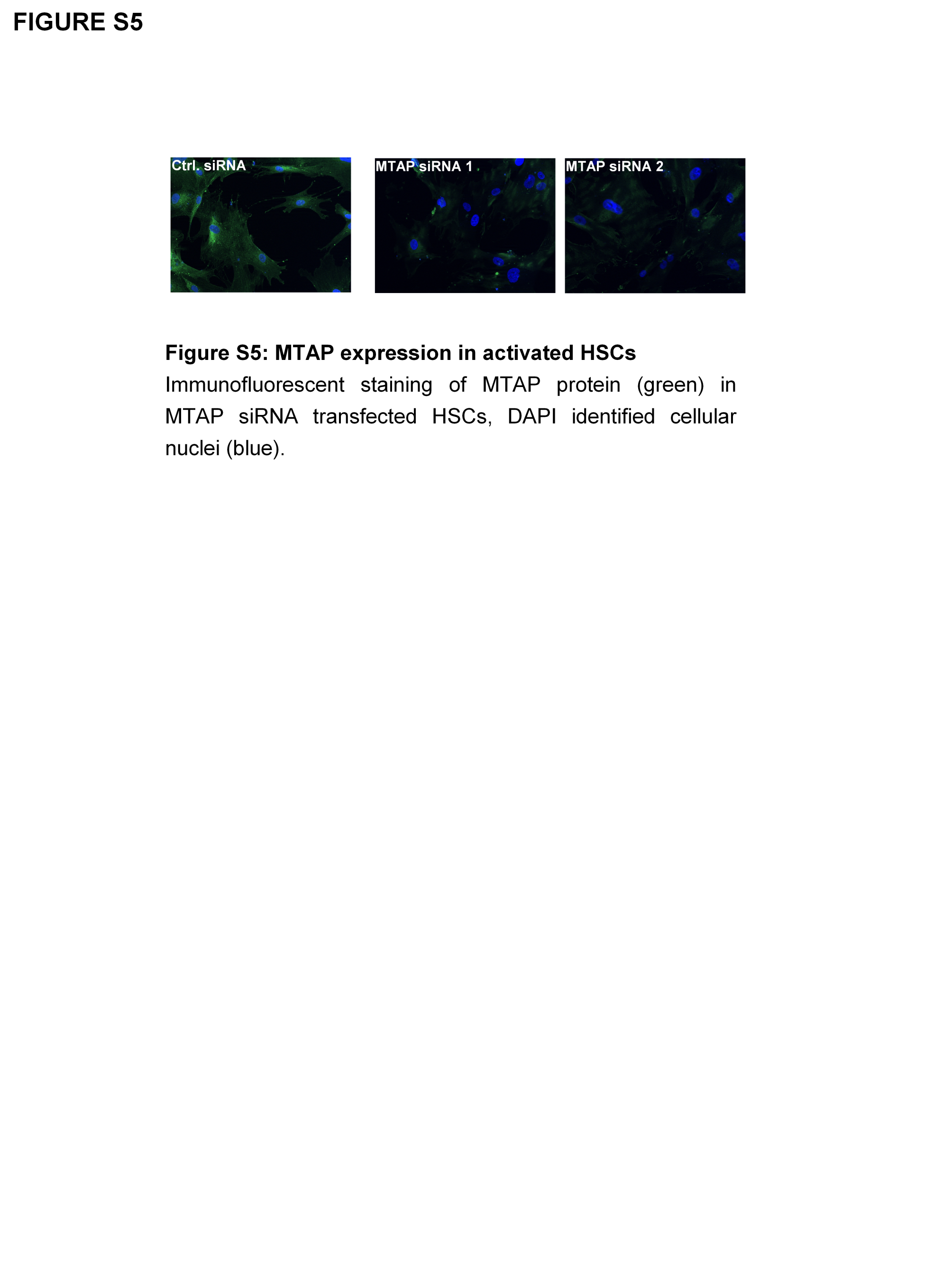

Supplement: Figure S5 — MTAP expression in activated HSCs. (TIF) [file pone.0080703.s005.tif]

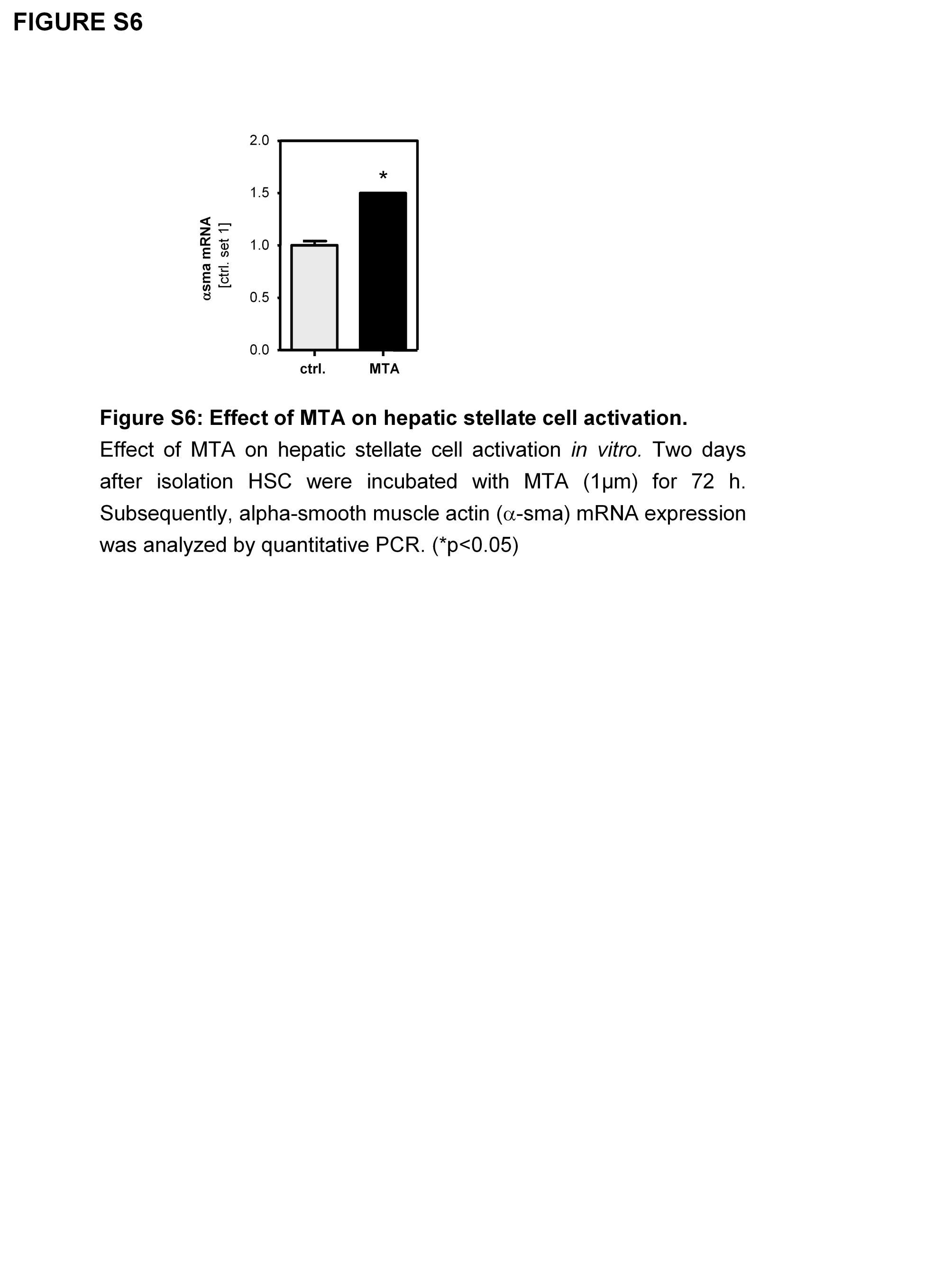

Supplement: Figure S6 — Effect of MTA on hepatic stellate cell activation. (TIF) [file pone.0080703.s006.tif]

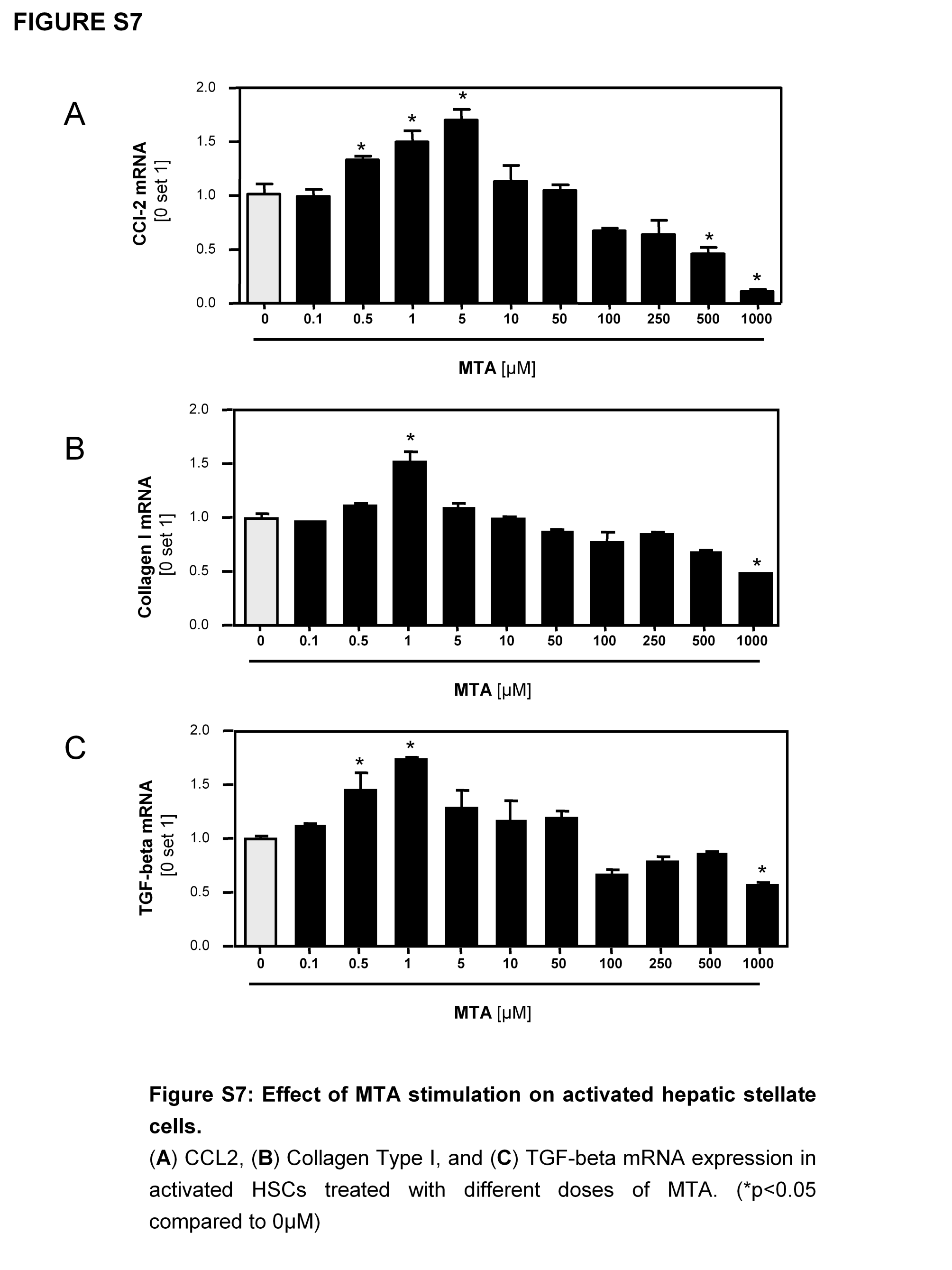

Supplement: Figure S7 — Effect of MTA stimulation on activated hepatic stellate cells. (TIF) [file pone.0080703.s007.tif]

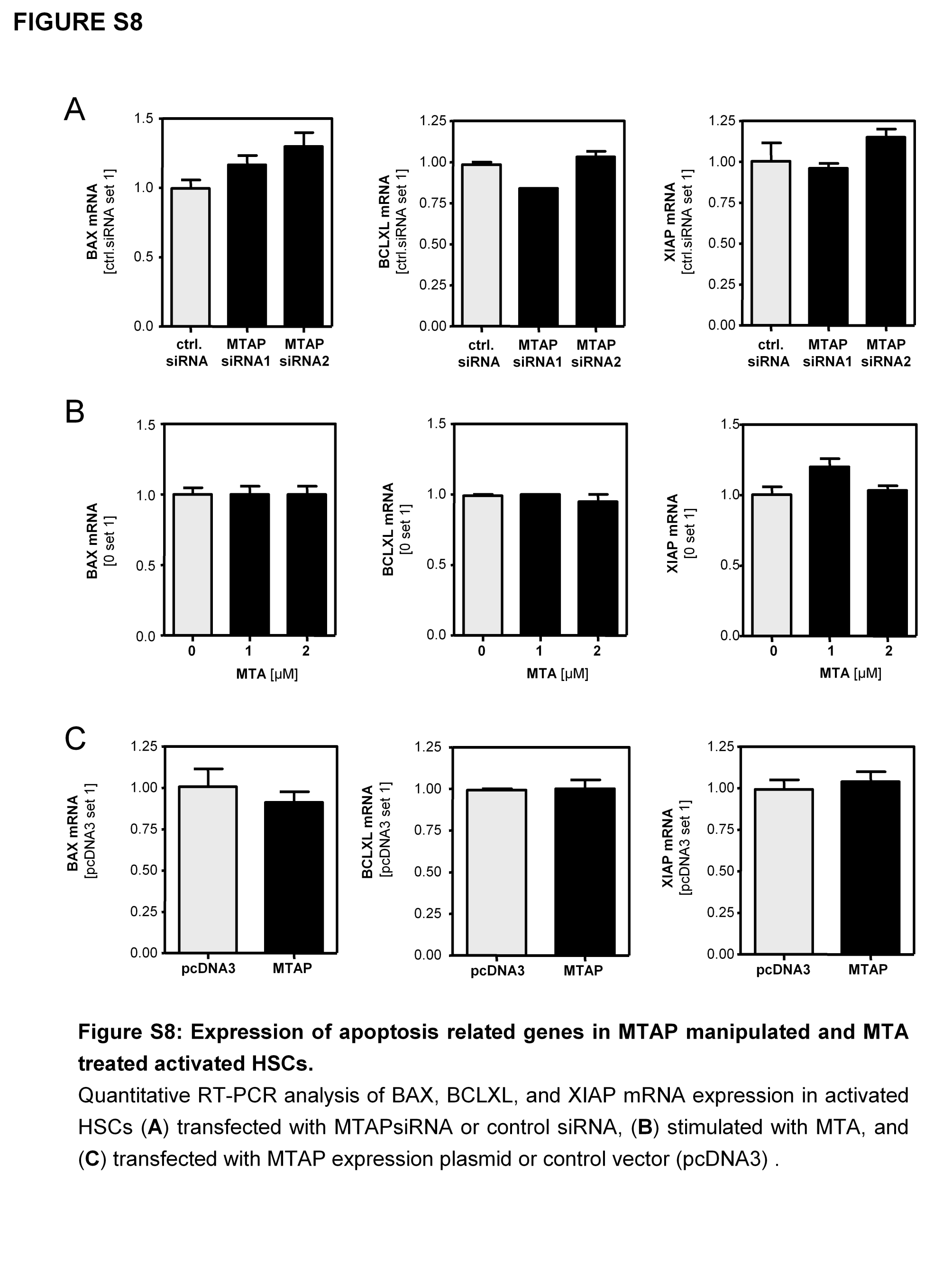

Supplement: Figure S8 — Expression of apoptosis related genes in MTAP manipulated and MTA treated activated HSCs. (TIF) [file pone.0080703.s008.tif]

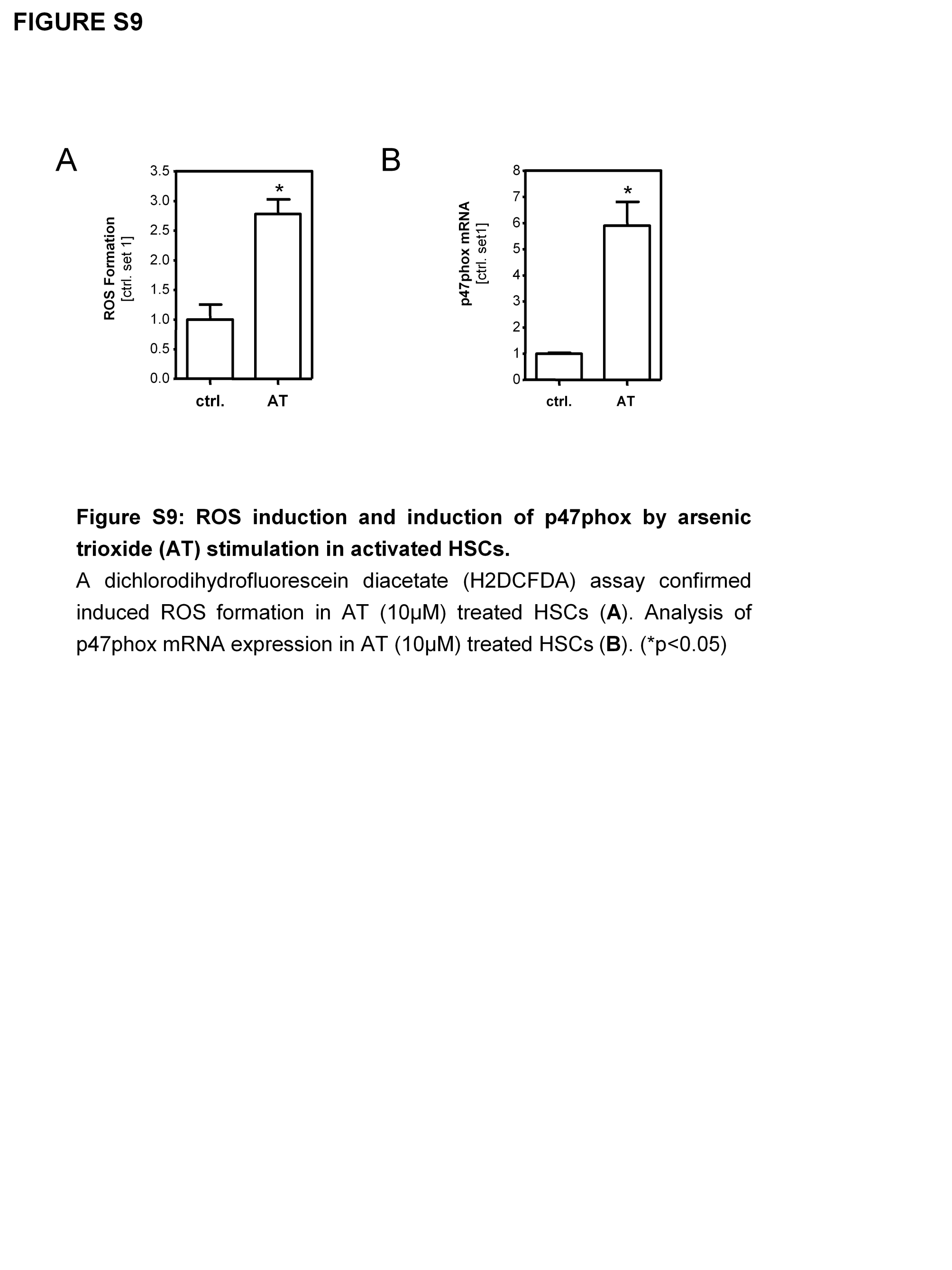

Supplement: Figure S9 — ROS induction and induction of p47phox by arsenic trioxide stimulation in activated HSCs. (TIF) [file pone.0080703.s009.tif]

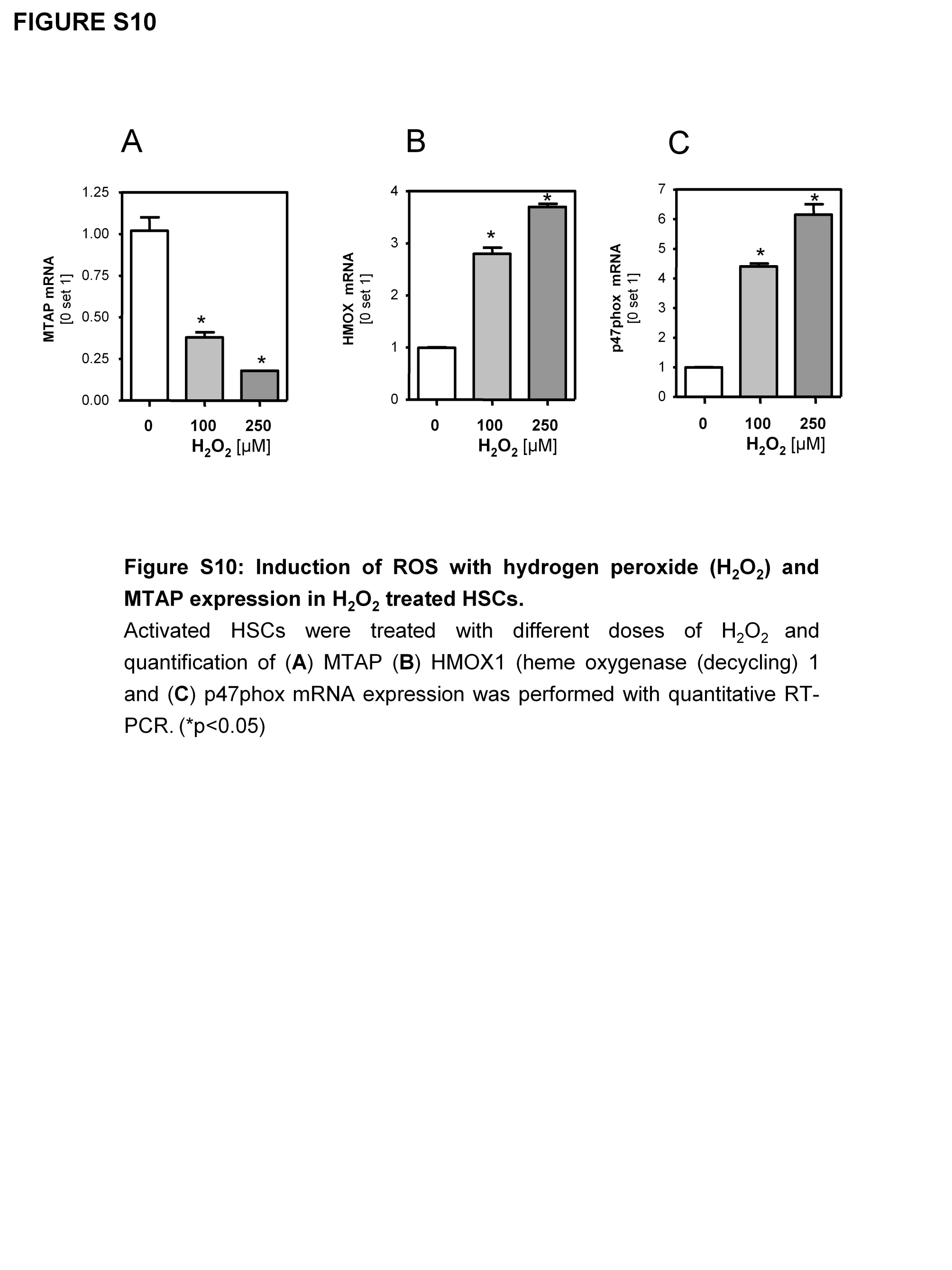

Supplement: Figure S10 — Induction of ROS with hydrogen peroxide and MTAP expression in H2O2 treated HSCs. (TIF) [file pone.0080703.s010.tif]

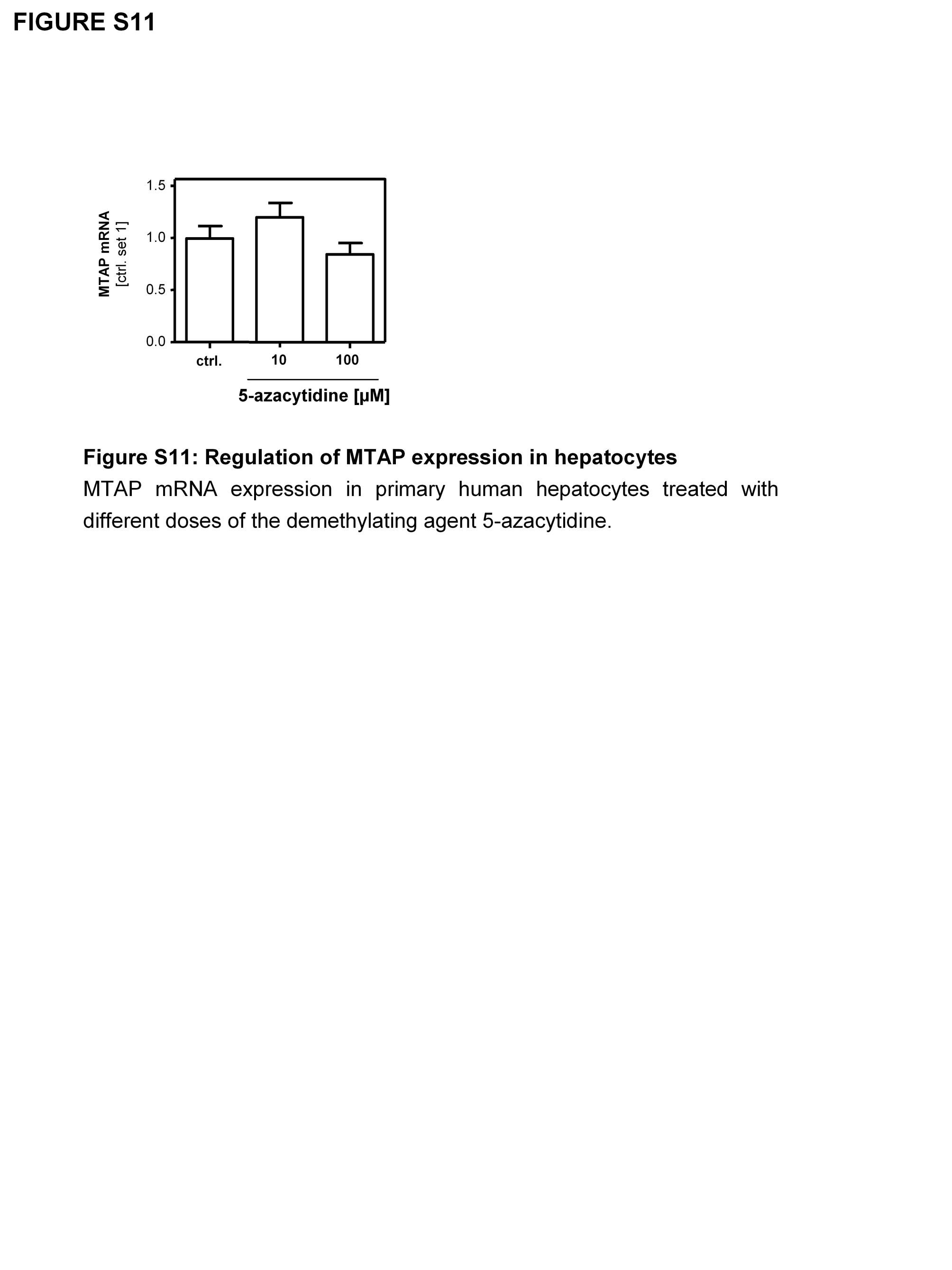

Supplement: Figure S11 — Regulation of MTAP expression in hepatocytes. (TIF) [file pone.0080703.s011.tif]
